# Designing a Network Proximity-Based Drug Repurposing Strategy for COVID-19

**DOI:** 10.3389/fcell.2020.545089

**Published:** 2020-10-06

**Authors:** Paola Stolfi, Luigi Manni, Marzia Soligo, Davide Vergni, Paolo Tieri

**Affiliations:** ^1^National Research Council (CNR), Institute for Applied Computing (IAC), Rome, Italy; ^2^National Research Council (CNR), Institute of Translational Pharmacology (IFT), Rome, Italy

**Keywords:** COVID-19, network medicine, drug repurposing, network-based, pharmacologic (drug) therapy

## Abstract

The ongoing COVID-19 pandemic still requires fast and effective efforts from all fronts, including epidemiology, clinical practice, molecular medicine, and pharmacology. A comprehensive molecular framework of the disease is needed to better understand its pathological mechanisms, and to design successful treatments able to slow down and stop the impressive pace of the outbreak and harsh clinical symptomatology, possibly via the use of readily available, off-the-shelf drugs. This work engages in providing a wider picture of the human molecular landscape of the SARS-CoV-2 infection via a network medicine approach as the ground for a drug repurposing strategy. Grounding on prior knowledge such as experimentally validated host proteins known to be viral interactors, tissue-specific gene expression data, and using network analysis techniques such as network propagation and connectivity significance, the host molecular reaction network to the viral invasion is explored and exploited to infer and prioritize candidate target genes, and finally to propose drugs to be repurposed for the treatment of COVID-19. Ranks of potential target genes have been obtained for coherent groups of tissues/organs, potential and distinct sites of interaction between the virus and the organism. The normalization and the aggregation of the different scores allowed to define a preliminary, restricted list of genes candidates as pharmacological targets for drug repurposing, with the aim of contrasting different phases of the virus infection and viral replication cycle.

## Introduction

The worldwide ongoing COVID-19 pandemic outnumbers 23.9M confirmed cases and a death toll above 819,000 (∼3.4% global case fatality rate), at the time of writing^1^ (Dong et al., 2020). Worse, in several densely populated countries, especially those in the South of the world, it is still difficult to forecast when a significant slowing down of the pace of the new infections will occur, and if, when and with what intensity a new global wave will arise. The ultimate goal in fighting a pandemic is to completely stop the spread, but slowing it down is also crucial, to mitigate otherwise devastating effects on health and socioeconomic systems on a local and global scale. Thus, it is necessary to interfere by every possible means with the natural, deadly flow of the outbreak, in order to reduce and flatten the epidemic curve and relieve the pressure on hospitals capacity (Qualls et al., 2017; Anderson et al., 2020).

In this perspective, aside all already implemented epidemiological, clinical and immunological measures and efforts, a deployment, via drug repurposing, of the vast, existing and potentially effective pharmacological arsenal is timely and needed, witnessed by the numerous ongoing clinical trials on several off-the shelf drugs (source: DrugBank)^2^. This work is committed to aid in the fight against the health consequences of the COVID-19 pandemic by providing a data-driven, viable drug repurposing approach.

In this study, we give account of the complexity of the molecular interactions and processes underlying the SARS-CoV-2 host response, and provide an integrated molecular picture to be exploited for a drug repurposing strategy. Such a picture includes the charting of the protein interaction map involving host genes that in the current state of knowledge have been observed to interact with SARS-CoV-2 viral proteins, and/or are considered critical in the host infection processes, also considering previous knowledge related to other relevant Coronaviruses. In the wider context of network medicine (Bauer-Mehren et al., 2011; Silverman and Loscalzo, 2017), the protein-protein interaction (PPI) framework provides a widely assessed and effective heuristic approach for the identification of disease genes (Taylor et al., 2009; Gustafsson et al., 2014; Tieri et al., 2019; Silverman et al., 2020). The complexity of the organism’s response to the viral invasion is mirrored by the wide variability of the clinical symptoms observed in patients, ranging from asymptomatic infections to extremely critical conditions, up to the death of the patient in around 3.4% of cases worldwide (see text footnote 1). With this study we therefore intended to expand the molecular landscape of the host proteins observed to directly interact with viral proteins (Gordon et al., 2020) to include actors who could be neglected when focusing only on the direct interactors set, and that could potentially prove to be important pharmacological targets to engage in order to propose an effective drug repurposing strategy aimed to improve the clinical outcome of the disease.

## Materials and Methods

**FIGURE 1:**
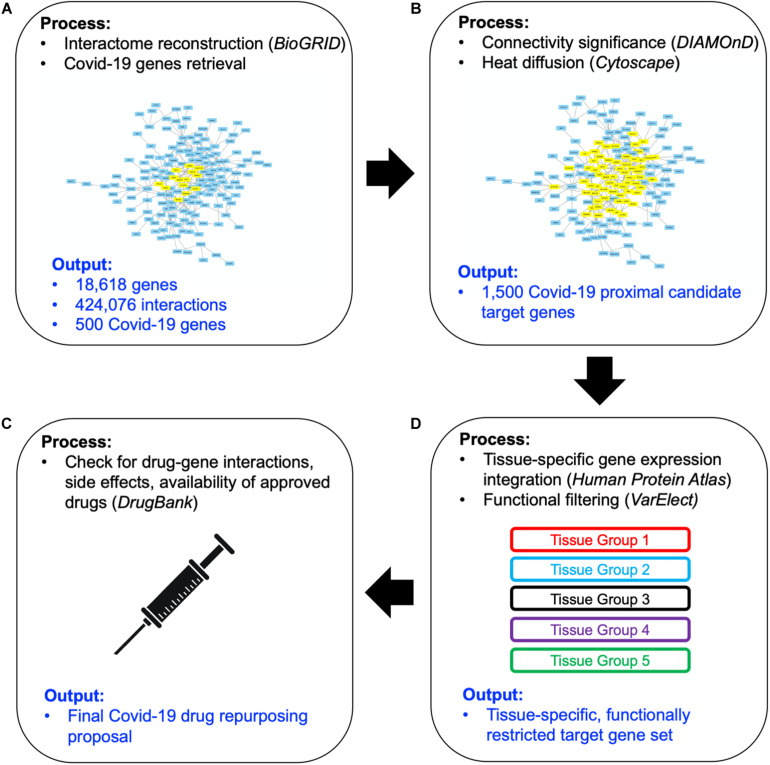
Scheme of the workflow adopted: starting from available human interactome data and the set of COVID-19 experimentally associated genes **(A)**, a network proximity approach (based on connectivity significance and heat diffusion) has been carried out to select genes that are proximal to the initial set of COVID seed genes **(B)**. Filtering via gene expression in specific tissues and association to the most common COVID-19 symptoms and phenotypes **(C)** allowed the design of the proposed drug repurposing strategy **(D)**.

The workflow of our approach has been sketched in Figure 1. Here we briefly describe each step of the method, providing detailed explanations in forthcoming subsections. We started by collecting updated human PPI data (Figure 1A) -from which a network of 18,618 human proteins and 424,076 binary interactions has been built- and SARS-CoV-2/Coronavirus/human PPI data, constituted by a set of 500 human genes potentially involved in the COVID-19 disease (see section “COVID-19 Associated Host Genes, Protein-Protein Interaction Data and Interactomes Reconstruction”). On such data, a network medicine approach has been applied by using connectivity significance and network diffusion algorithms in order to provide a COVID-19 “proximity” or “involvement” gene ranking (Figure 1B, details in section “Connectivity Significance” and “Network Diffusion”).

**FIGURE 2:**
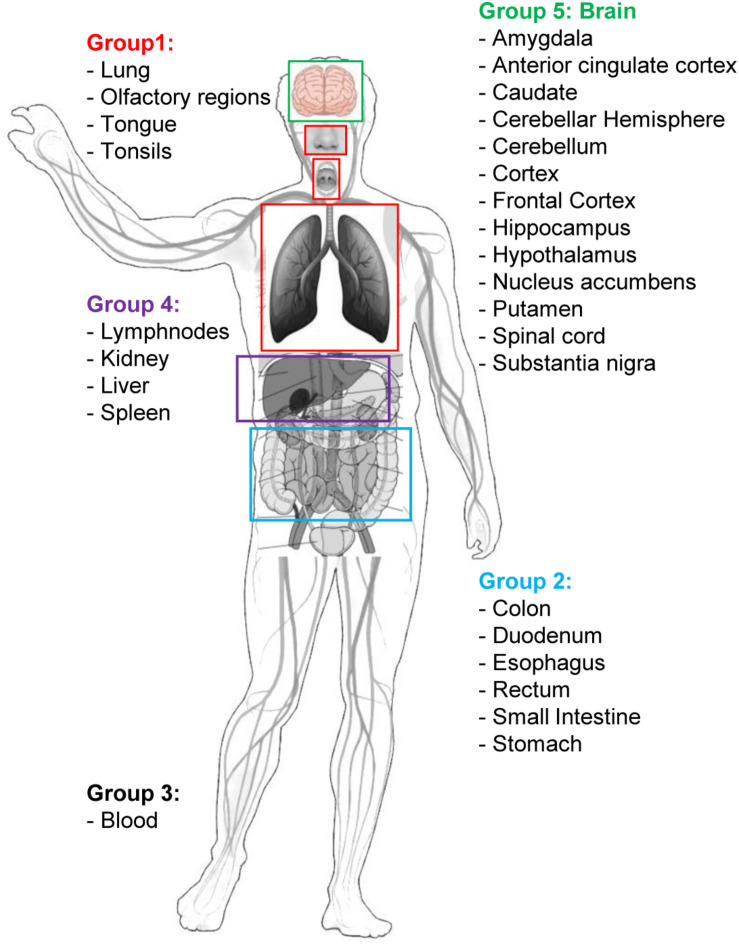
Graphical sketch showing the selected five groups of organs/tissues representative of potential sites of interaction between SARS-CoV-2 and the organism. Group 1: respiratory tract tissues; Group 2: organs of the digestive system; Group 3: blood cells; Group 4: filtering organs; Group 5: brain areas. Group-specific gene expression data have been retrieved by the Human Protein Atlas web portal (www.proteinatlas.org).

The top 1,000 genes in the proximity ranking added to the original 500 Sars-CoV-2 related genes gives the final dataset of 1,500 mostly involved proteins in the COVID-19 disease. In order to further refine the selected list of genes, their gene expression levels in COVID-19-relevant tissues have been investigated (Figure 1C). The human tissues mostly involved in the COVID-19 infection have been identified and divided into five groups (see Figure 2 and section “Gene Expression Data”). The genes that are not expressed in those tissues have been excluded. The remaining genes, for each tissue group, are ranked based on the most common COVID-19 symptoms. The rankings have been provided through VarElect functional filtering, whose details have been discussed in section “Functional Analysis,” and they have been aggregated (see section “Rank Aggregation”), so that a restricted ranked list has been considered. Finally (Figure 1D), the proposed drug repositioning strategy was designed and implemented via dedicated drug-gene interaction information (see section “Design of Drug Repositioning Strategy via Drug-Gene Interaction Data”).

### COVID-19 Associated Host Genes, Protein-Protein Interaction Data and Interactomes Reconstruction

Protein-protein interaction data for interactome reconstruction have been retrieved from the BioGRID (Oughtred et al., 2019), one of the most comprehensive interaction repositories with freely provided data compiled through manual curation efforts, currently containing more than 1.7 million protein and genetic interactions from major model organism species, including Homo sapiens. The repository provides both the whole human-only interactome, as well as, in the effort to provide valuable data to fight the pandemic, the SARS-CoV-2/human protein interaction dataset, derived from several sources as described on the dedicated BioGRID webpage^3^. For this study, the latest version available at the time of the analysis of the human interactome, and of COVID-19-associated host genes, i.e., version 3.5.186 (.tab2 and .tab3 format types) have been used. The dataset includes 338 human proteins interacting with SARS-CoV-2 [i.e., the genes identified by the seminal work of Gordon and colleagues (Gordon et al., 2020)], 47 human proteins considered critical for the virus host entry and response, and further 115 proteins experimentally observed to interact with other, SARS-relevant Coronaviruses, finally totaling 500 involved human genes (Supplementary Table S1). The reason for including the last 115 genes is found in the fact that it is known that there is marked similarity and a close relationship between SARS-CoV-2 and SARS-CoVs or SARS-like bat CoVs (Wu A. et al., 2020), similarities that could play a relevant role when comparing the host tropism and transmission features of the SARS-CoV-2 and SARS-CoV and that are thus worthy of investigation. Besides these considerations, and despite the efforts in experimental PPI mapping, it is also known that the number of missing interactions greatly exceeds the number of experimentally detected interactions (Kovács et al., 2019). In this perspective, these further viral-human interactions related to other Coronaviruses provided by BioGRID in the same dataset represent a very significant information from the heuristic point of view, partly due to structural similarities.

The whole human interactome has been gathered from BioGRID data as well (BIOGRID-ORGANISM-3.5.186.tab3.zip), and the largest connected component (LCC) has been extracted to undergo network analysis, consisting of 18,618 genes and 424,076 unique pairwise interactions among them (Supplementary File S1).

### Connectivity Significance

The concept of connectivity significance, originally proposed by Ghiassian et al. (2015), has been used to uncover genes associated with a particular path phenotype, via the observation that proteins associated to specific diseases show peculiar patterns of interaction among each other, patterns that in turn help in the identification of neighborhoods not previously associated to the disease. An efficient algorithm (a.k.a. DIAMOnD, DIseAse MOdule Detection) to compute this measure is publicly available (Ghiassian et al., 2015), and it has been used to rank the genes in the interactome showing the highest connectivity significance with respect to the 500 COVID-19-associated seed genes (in Supplementary Table S2 were reported the first 200 ranked genes).

### Network Diffusion

Network diffusion (or network propagation) is a methodology able to identify those genes which are proximal to a starting list of seed genes by using network topology (and optionally other features). In network medicine it can be used to identify genes and genetic modules that underlie human diseases (Mosca et al., 2014; Cowen et al., 2017; Sumathipala et al., 2019) or to identify causal paths linking mutations to expression regulators, or to discover significantly mutated subnetworks in cancer (Vandin et al., 2011; Paull et al., 2013). The methodology exploits the concept of heat diffusion, i.e., how the heat distribution spreads over time in a medium, here consisting of the PPI network, as it flows from nodes where it is higher toward nodes where it is lower according to the diffusion coefficient and their mutual connections. In practice, starting with an arbitrary subset of seed nodes (e.g., genes associated with a disease), a diffusion algorithm is applied to the initial values assigned to the seed nodes that propagate through the network according to its topology. Fixing a stopping time for the diffusion algorithm, the final distribution of the propagated values generates a proximity ranking that can be used to identify a subset of genes that are closely associated to the selected seed genes. The Cytoscape network analysis platform (Shannon et al., 2003), version 3.7, and the Cytoscape-embedded function “Diffuse,” based on a heat diffusion algorithm, have been used for the analysis (Carlin et al., 2017). The diffusion algorithm has been run considering as seed genes the 500 COVID-19-associated human genes with initial heat *h*_s_*(0)* = 1; non-seed genes have been set with initial heat *h*_ns_*(0) = 0*. The heat diffusion has been observed at the following times *t*: 0.002, 0.005, 0.01, 0.02, 0.05 (arbitrary algorithm diffusion time units; Supplementary Table S3), and the quantities of heat in non-seed genes *h*_ns_*(t)* have been computed. The appropriate time has been identified by considering, for each time *t*, the intersection of the most significant genes obtained via the DIAMOnD algorithm and the most relevant genes in the diffusion process, i.e., the ones with highest *h*_ns_*(t)* values, and selecting the time showing the largest overlap, that turned out to be *t* = 0.005. More in detail, we considered the overlap of the top 200 genes obtained via the DIAMOnD algorithm with the top 1000 genes obtained via the heat diffusion algorithm at each stopping time. The combination of the two methods, the heat diffusion that favors genes well-connected to the seed genes or with high degrees, and the DIAMOnD that privileged those genes that are well-connected to the set of the seed genes, generates a proximity ranking of topologically well-connected genes to the COVID-19-associated genes. Moreover, since the overlap in the intersection is about 50%, the number of genes that is surely well-connected to the seed genes is very significant.

### Rank Aggregation

Rank aggregation deals with the aggregation of several lists of preferences obtained from different methodologies. It is very useful in all those situations in which preferences can be set according to several features, none of them prevailing on the others. This is actually our case with different lists of best genes associated with the different preferences, none being preferred over the others. Many methods have been proposed in literature to aggregate rank, they are mainly divided into three groups, namely heuristic algorithms, methods based on Markov chains and stochastic optimization methods, see Lin (2010) for a detailed overview. The most suitable method in this particular situation turned out to be a stochastic optimization method. Namely, a new ranking is obtained through an optimization problem whose objective is to minimize the distance between the new ranking and all the others. This approach usually considers two distances, the L1, also known in the rank aggregation literature as Spearman’s distance, and the Kendall distance. The main difference between these two measures is that the first one considers the distances between the different scores of the genes in the different lists of preferences, while the second one takes into account the partial order of the ranking counting the number of pairwise discordance between two lists of preferences. The optimization has been carried out using the L1 distance over the list of preference obtained from the VarElect tool detailed in section “Functional Analysis.”

### Gene Expression Data

Human tissue-specific gene expression data have been retrieved by the Human Protein Atlas web portal^4^ (Uhlén et al., 2015). The Tissue Atlas includes information about the expression profiles of human genes on mRNA and protein level. The protein data covers 15,313 genes (78%) for which there are antibodies available. The mRNA expression data are derived from RNA-seq of 37 different healthy individuals. Genes expressed in 5 organs and tissue groups, representative of potential sites of interaction between SARS-CoV-2 and the organism, were first selected (Table 1 and Figure 2), based on up-to-date information^5^. Indeed, it is actually recognized that, beside its impact on the respiratory system, SARS-CoV-2 induces multi-organ dysfunctions (Bal et al., 2020; Wu T. et al., 2020) indicating a potential virus-host interaction extended to several organs/systems. Respiratory tract tissues (lungs, tongue, tonsils, and olfactory epithelium) were included in group 1. In group 2, organs and tissues of the digestive system (stomach, esophagus, colon, duodenum, small intestine and rectum) were included. Groups 1 and 2 are therefore representative of the highest probability of virus-host interaction, affecting the epithelial cells (Cong and Ren, 2014). All blood cells were included in group 3. In group 4 the filtering organs and tissues (spleen, liver, lymph nodes, and kidney) were included. Finally, all brain areas for which RNA expression data were available in Protein Atlas (Amygdala, Basal Ganglia, Cerebellum, Cerebral Cortex, Hippocampus, Hypothalamus, Midbrain, Olfactory region, Pons and Medulla, and Thalamus) were included in group 5. The need to include tissues belonging to the nervous system in the analysis derives from the emerging evidence of a specific involvement of the latter in the development of symptoms currently named Neuro-COVID (Ahmad and Rathore, 2020; Helms et al., 2020; Mao et al., 2020). For each group, genes with an expression level <2 (for details about normalized RNA expression data see “Normalization of transcriptomics data” section in the Protein Atlas web portal)^6^ in all tissues/organs belonging to each group were excluded from the analysis. In each of the groups, the 1,500 mostly involved proteins in the COVID-19 disease were selected according to their expression level and used for the functional analysis through the VarElect tool (Stelzer et al., 2016), see section “Functional Analysis” for details.

**TABLE 1:** Groups of tissue/organs matched with disease phenotypes **(A)** and number of target genes selected as potential candidates for drug repurposing by VarElect aggregate and single group ranks **(B)**.

(A) Groups of tissue/organs matched with disease phenotypes			

Group # ID	Organ systems	Organs and tissues	Disease phenotypes (symptoms or disease manifestations)
Group 1	Respiratory tract	Lungs, tongue, tonsils, olfactory epithelium	“Fever” OR “cough” OR “pneumonia” OR “dyspnea” OR “pain” OR “hemoptysis” OR “sore throat” OR “chills” OR “inflammation”

Group 2	Digestive system	Stomach, esophagus, colon, duodenum, small intestine and rectum	“Fever” OR “diarrhea” OR “pain” OR “nausea” OR “vomiting” OR “inflammation”

Group 3	Blood cells	n/a	“Fever” OR “chills” OR “inflammation” OR “hemorrhagic”

Group 4	Filtering organs	Spleen, liver, lymph nodes, kidney	“Fever” OR “cough” OR “diarrhea” OR “pain” OR “nausea” OR “vomiting” OR “chills” OR “inflammation” OR “hemorrhagic”

Group 5a	Brain areas	Amygdala, Basal Ganglia, Cerebellum, Cerebral Cortex, Hippocampus, Hypothalamus, Midbrain, Olfactory region, Pons and Medulla, Thalamus	“Dizziness” OR “headache” OR “consciousness” OR “encephalopathy” OR “encephalitis” OR “seizures” OR “stroke” OR “delirium”

Group 5b	Brain areas	Amygdala, Basal Ganglia, Cerebellum, Cerebral Cortex, Hippocampus, Hypothalamus, Midbrain, Olfactory region, Pons and Medulla, Thalamus	“Ageusia” OR “dysgeusia” OR “hypogeusia” OR “anosmia” OR “hyposmia” OR “myalgia” OR “myelitis” OR “pain” OR “Guillain-Barre”

**(B) Number of target genes selected as potential candidates for drug repurposing by VarElect aggregate and single group ranks**			

**Group # ID**	**Selected gene targets (potential candidates for drug repurposing)**	**Selected genes**	**Selection of the first 15 genes**

Group 1+2+4 (G124)	101	See Supplementary Table S11	See Table 2

Group 1+2+3+4+5 (G12345)	99	See Supplementary Table S12	See Table 3

Group 3 (G3)	20	See Supplementary Table S13	See Table 4

Group 5a (G5a)	20	See Supplementary Table S14	See Table 5

Group 5b (G5b)	20	See Supplementary Table S15	See Table 6

### Functional Analysis

We took advantage from the VarElect tool, a comprehensive phenotype-dependent gene prioritizer, based on the widely used GeneCards, which helps in identifying causal gene-phenotype associations with extensive evidence (Stelzer et al., 2016). The sets of COVID-host interacting genes, selected for each group of tissue/organs, were matched with disease phenotypes (symptoms or disease manifestations) that were considered peculiar to each group of organs/tissues (Table 1). Accordingly, for group 1 the phenotype query: “fever” OR “cough” OR “pneumonia” OR “dyspnea” OR “pain” OR “hemoptysis” OR “sore throat” OR “chills” OR “inflammation” was used (Supplementary Table S5). Group 2 was analyzed for the phenotype query: “fever” OR “diarrhea” OR “pain” OR “nausea” OR “vomiting” OR “inflammation” (Supplementary Table S6). Group 3 was analyzed for the phenotypes: “fever” OR “chills” OR “inflammation” OR “hemorrhagic” (Supplementary Table S7). The phenotype query for group 4 was: “fever” OR “cough” OR “diarrhea” OR “pain” OR “nausea” OR “vomiting” OR “chills” OR “inflammation” OR “hemorrhagic” (Supplementary Table S8). For group 5 (brain tissues) 2 sets of disease-related phenotypes were used in separate VarElect analyses, accounting for the reported neurological symptoms related to the central nervous system (group 5a, phenotype query: “dizziness” OR “headache” OR “consciousness” OR “encephalopathy” OR “encephalitis” OR “seizures” OR “stroke” OR “delirium,” Supplementary Table S9) or the peripheral nervous system (group 5b, phenotype query: “ageusia” OR “dysgeusia” OR “hypogeusia” OR “anosmia” OR “hyposmia” OR “myalgia” OR “myelitis” OR “pain” OR “Guillain-Barre,” Supplementary Table S10) (Ahmad and Rathore, 2020; Lahiri and Ardila, 2020; Tsivgoulis et al., 2020).

The VarElect scores related to each tissue group have been normalized so that they can be compared and used in a rank aggregation procedure. In particular, we considered two rank aggregation, the first by aggregating groups 1,2, and 4 (G124) and the second by aggregating groups 1,2,3,4, and 5 (G12345).

The VarElect analysis on single and aggregate groups allowed the selection of 260 (arbitrary cutoff, subject to extension in forthcoming analysis) gene targets potential candidates for drug repurposing (complete lists in Supplementary Tables S11–S15, selection of the first 15 genes for each aggregate or single group ranks in Tables 2–6). In particular, 101 genes were selected for the aggregate rank G124 (Supplementary Table S11), 99 genes were selected for the aggregate rank G12345 (Supplementary Table S12), and 20 genes were selected for each of the single analysis performed on group 3 (G3 blood cells, Supplementary Table S13), group 5a (G5a brain, VarElect analysis related to the central nervous system, Supplementary Table S14), and group 5b (G5b brain, VarElect analysis related to the peripheral nervous system, Supplementary Table S15).

**TABLE 2:** VarElect aggregated score obtained analyzing groups 1, 2, and 4 (G124; 15 top-ranking genes).

Aggregated score (normalized)	Gene	Gene description	Protein class	Biological process	Molecular function	Disease involvement	Subcellular Location
0.999501151	TNF	Tumor necrosis factor	Cancer-related genes, Candidate cardiovascular disease genes, Disease related genes, FDA approved drug targets, Plasma proteins, Predicted intracellular proteins, Predicted secreted proteins		Cytokine	Cancer-related genes, FDA approved drug targets	Secreted
0.624534796	TNFRSF1A	TNF receptor superfamily member 1A	Cancer-related genes, Candidate cardiovascular disease genes, CD markers, Disease related genes, FDA approved drug targets, Plasma proteins, Predicted membrane proteins, Predicted secreted proteins	Apoptosis, Host-virus interaction	Receptor	Amyloidosis, Cancer-related genes, Disease mutation, FDA approved drug targets	
0.529423274	TP53	Tumor protein p53	Cancer-related genes, Disease related genes, Plasma proteins, Potential drug targets, Predicted intracellular proteins, Transcription factors, Transporters	Apoptosis,Biological rhythms, Cell cycle, Host-virus interaction, Necrosis, Transcription, Transcription regulation	Activator, DNA-binding, Repressor	Cancer-related genes, Disease mutation, Li-Fraumeni syndrome, Tumor suppressor	Nucleoplasm
0.479435236	NLRP3	NLR family pyrin domain containing 3	Cancer-related genes, Disease related genes, Predicted intracellular proteins	Immunity, Inflammatory response, Innate immunity, Transcription, Transcription regulation	Activator	Amyloidosis, Cancer-related genes, Deafness, Disease mutation, Non-syndromic deafness	
0.406134194	TGFB1	Transforming growth factor beta 1	Cancer-related genes, Candidate cardiovascular disease genes, Disease related genes, Plasma proteins, Predicted intracellular proteins, Predicted secreted proteins		Growth factor, Mitogen	Cancer-related genes, Disease mutation	Golgi apparatus, secreted
0.404538712	EGFR	Epidermal growth factor receptor	Cancer-related genes, Disease related genes, Enzymes, FDA approved drug targets, Plasma proteins, Predicted intracellular proteins, Predicted membrane proteins, Predicted secreted proteins, RAS pathway related proteins	Host-virus interaction	Developmental protein, Host cell receptor for virus entry, Kinase, Receptor, Transferase, Tyrosine-protein kinase	Cancer-related genes, Disease mutation, FDA approved drug targets, Proto-oncogene	Plasma membrane
0.393038016	ICAM1	Intercellular adhesion molecule 1	Cancer-related genes, Candidate cardiovascular disease genes, CD markers, FDA approved drug targets, Plasma proteins, Predicted intracellular proteins, Predicted membrane proteins	Cell adhesion, Host-virus interaction	Host cell receptor for virus entry, Receptor	Cancer-related genes, FDA approved drug targets	Plasma membrane
0.37555677	FAS	Fas cell surface death receptor	Cancer-related genes, Candidate cardiovascular disease genes, CD markers, Disease related genes, Predicted membrane proteins, Predicted secreted proteins	Apoptosis	Calmodulin-binding, Receptor	Cancer-related genes, Disease mutation	Plasma membrane
0.352296	STAT3	Signal transducer and activator of transcription 3	Cancer-related genes, Disease related genes, Plasma proteins, Predicted intracellular proteins, Transcription factors	Host-virus interaction, Transcription, Transcription regulation	Activator, DNA-binding	Cancer-related genes, Diabetes mellitus, Disease mutation, Dwarfism	Nucleoplasm, cytosol
0.337175731	STAT1	Signal transducer and activator of transcription 1	Disease related genes, Plasma proteins, Predicted intracellular proteins, Transcription factors	Antiviral defense, Host-virus interaction, Transcription, Transcription regulation	Activator, DNA-binding	Disease mutation	Nucleoplasm, cytosol
0.336625898	KRAS	KRAS proto-oncogene, GTPase	Cancer-related genes, Disease related genes, Predicted intracellular proteins, RAS pathway related proteins			Cancer-related genes, Cardiomyopathy, Deafness, Disease mutation, Ectodermal dysplasia, Mental retardation, Proto-oncogene	Cytosol
0.324061896	CTNNB1	Catenin beta 1	Cancer-related genes, Disease related genes, Plasma proteins, Predicted intracellular proteins	Cell adhesion, Host-virus interaction, Neurogenesis, Transcription, Transcription regulation, Wnt signaling pathway	Activator	Cancer-related genes, Disease mutation, Mental retardation	Plasma membrane
0.315947057	AKT1	AKT serine/threonine kinase 1	Cancer-related genes, Disease related genes, Enzymes, Potential drug targets, Predicted intracellular proteins, RAS pathway related proteins	Apoptosis, Carbohydrate metabolism, Glucose metabolism, Glycogen biosynthesis, Glycogen metabolism, Neurogenesis, Sugar transport, Translation regulation, Transport	Developmental protein, Kinase, Serine/threonine-protein kinase, Transferase	Cancer-related genes, Disease mutation, Proto-oncogene	Nucleoplasm
0.314911716	CCND1	Cyclin D1	Cancer-related genes, Disease related genes, FDA approved drug targets, Predicted intracellular proteins	Cell cycle, Cell division, DNA damage, Transcription, Transcription regulation	Cyclin, Repressor	Cancer-related genes, FDA approved drug targets, Proto-oncogene	Nucleoplasm
0.312644606	ERBB2	Erb-b2 receptor tyrosine kinase 2	Cancer-related genes, CD markers, Disease related genes, Enzymes, FDA approved drug targets, Plasma proteins, Predicted intracellular proteins, Predicted membrane proteins	Transcription, Transcription regulation	Activator, Kinase, Receptor, Transferase, Tyrosine-protein kinase	Cancer-related genes, FDA approved drug targets	Plasma membrane

**TABLE 3:** VarElect aggregated score obtained analyzing groups 1, 2, 3, 4, and 5 (G12345; 15 top-ranking genes).

Aggregated score (normalized)	Gene	Gene description	Protein class	Biological process	Molecular function	Disease involvement	Subcellular Location
0.999992851	TNF	Tumor necrosis factor	Cancer-related genes, Candidate cardiovascular disease genes, Disease related genes, FDA approved drug targets, Plasma proteins, Predicted intracellular proteins, Predicted secreted proteins		Cytokine	Cancer-related genes, FDA approved drug targets	
0.646667342	TNFRSF1A	TNF receptor superfamily member 1A	Cancer-related genes, Candidate cardiovascular disease genes, CD markers, Disease related genes, FDA approved drug targets, Plasma proteins, Predicted membrane proteins, Predicted secreted proteins	Apoptosis, Host-virus interaction	Receptor	Amyloidosis, Cancer-related genes, Disease mutation, FDA approved drug targets	
0.469465581	TP53	Tumor protein p53	Cancer-related genes, Disease related genes, Plasma proteins, Potential drug targets, Predicted intracellular proteins, Transcription factors, Transporters	Apoptosis, Biological rhythms, Cell cycle, Host-virus interaction, Necrosis, Transcription, Transcription regulation	Activator, DNA-binding, Repressor	Cancer-related genes, Disease mutation, Li-Fraumeni syndrome, Tumor suppressor	Nucleoplasm
0.415888546	TGFB1	Transforming growth factor beta 1	Cancer-related genes, Candidate cardiovascular disease genes, Disease related genes, Plasma proteins, Predicted intracellular proteins, Predicted secreted proteins		Growth factor, Mitogen	Cancer-related genes, Disease mutation	Golgi apparatus, Cytosol
0.413794221	NLRP3	NLR family pyrin domain containing 3	Cancer-related genes, Disease related genes, Predicted intracellular proteins	Immunity, Inflammatory response, Innate immunity, Transcription, Transcription regulation	Activator	Amyloidosis, Cancer-related genes, Deafness, Disease mutation, Non-syndromic deafness	
0.374658935	FAS	Fas cell surface death receptor	Cancer-related genes, Candidate cardiovascular disease genes, CD markers, Disease related genes, Predicted membrane proteins, Predicted secreted proteins	Apoptosis	Calmodulin-binding, Receptor	Cancer-related genes, Disease mutation	Plasma membrane
0.332023563	STAT1	Signal transducer and activator of transcription 1	Disease related genes, Plasma proteins, Predicted intracellular proteins, Transcription factors	Antiviral defense, Host-virus interaction, Transcription, Transcription regulation	Activator, DNA-binding	Disease mutation	Nucleoplasm, Cytosol
0.330861395	ICAM1	Intercellular adhesion molecule 1	Cancer-related genes, Candidate cardiovascular disease genes, CD markers, FDA approved drug targets, Plasma proteins, Predicted intracellular proteins, Predicted membrane proteins	Cell adhesion, Host-virus interaction	Host cell receptor for virus entry, Receptor	Cancer-related genes, FDA approved drug targets	Plasma membrane, Cytosol
0.325734154	STAT3	Signal transducer and activator of transcription 2	Disease related genes, Predicted intracellular proteins, Transcription factors	Antiviral defense, Host-virus interaction, Transcription, Transcription regulation	Activator, DNA-binding		Plasma membrane, Cytosol
0.285387576	AKT1	Signal transducer and activator of transcription 3	Cancer-related genes, Disease related genes, Plasma proteins, Predicted intracellular proteins, Transcription factors	Host-virus interaction, Transcription, Transcription regulation	Activator, DNA-binding	Cancer-related genes, Diabetes mellitus, Disease mutation, Dwarfism	Nucleoplasm
0.280849434	CTNNB1	Catenin beta 1	Cancer-related genes, Disease related genes, Plasma proteins, Predicted intracellular proteins	Cell adhesion, Host-virus interaction, Neurogenesis, Transcription, Transcription regulation, Wnt signaling pathway	Activator	Cancer-related genes, Disease mutation, Mental retardation	Plasma membrane
0.267915871	SOD1	Superoxide dismutase 1	Cancer-related genes, Disease related genes, Enzymes, Plasma proteins, Potential drug targets, Predicted intracellular proteins		Antioxidant, Oxidoreductase	Amyotrophic lateral sclerosis, Cancer-related genes, Disease mutation, Neurodegeneration	Nucleoplasm, Plasma membrane
0.267304947	SPP1	Secreted phosphoprotein 1	Cancer-related genes, Plasma proteins, Predicted secreted proteins	Biomineralization, Cell adhesion	Cytokine	Cancer-related genes	Golgi apparatus
0.266717153	KRAS	KRAS proto-oncogene, GTPase	Cancer-related genes, Disease related genes, Predicted intracellular proteins, RAS pathway related proteins			Cancer-related genes, Cardiomyopathy, Deafness, Disease mutation, Ectodermal dysplasia, Mental retardation, Proto-oncogene	
0.25614674	PTEN	Phosphatase and tensin homolog	Cancer-related genes, Disease related genes, Enzymes, Potential drug targets, Predicted intracellular proteins, Predicted secreted proteins	Apoptosis, Lipid metabolism, Neurogenesis	Hydrolase, Protein phosphatase	Autism spectrum disorder, Cancer-related genes, Disease mutation, Tumor suppressor	Nucleoplasm, Cytosol

**TABLE 4:** VarElect aggregated score obtained analyzing group 3 (G3; 15 top-ranking genes).

Normalized score	Gene	Gene description	Protein class	Biological process	Molecular function	Disease involvement	Subcellular Location
1	TBK1	TANK Binding Kinase 1	Disease related genes, Enzymes, Potential drug targets, Predicted intracellular proteins, RAS pathway related proteins	Antiviral defense, Host-virus interaction, Immunity, Innate immunity	Kinase, Serine/threonine-protein kinase, Transferase	Amyotrophic lateral sclerosis, Disease mutation, Glaucoma, Neurodegeneration	Nucleoplasm, Vesicles
0.855967078	TNFAIP3	TNF Alpha Induced Protein 3	Cancer-related genes, Disease related genes, Enzymes, Plasma proteins, Potential drug targets, Predicted intracellular proteins	Apoptosis, Inflammatory response, Ubl conjugation pathway	DNA-binding, Hydrolase, Multifunctional enzyme, Protease, Thiol protease, Transferase	Cancer-related genes, Disease mutation	Vesicles
0.695473251	RANBP2	RAN Binding Protein 2	Cancer-related genes, Disease related genes, Plasma proteins, Potential drug targets, Predicted intracellular proteins, Transporters	mRNA transport, Protein transport, Translocation, Transport, Ubl conjugation pathway	RNA-binding, Transferase	Cancer-related genes	Nuclear membrane, Vesicles
0.582167353	APP	Amyloid Beta Precursor Protein	Disease related genes, FDA approved drug targets, Plasma proteins, Predicted intracellular proteins, Predicted membrane proteins, Transporters	Apoptosis, Cell adhesion, Endocytosis, Notch signaling pathway	Heparin-binding, Protease inhibitor, Serine protease inhibitor	Alzheimer disease, Amyloidosis, Disease mutation, FDA approved drug targets, Neurodegeneration	Golgi apparatus; Vesicles
0.573662551	PPARG	Peroxisome Proliferator Activated Receptor Gamma	Cancer-related genes, Disease related genes, FDA approved drug targets, Nuclear receptors, Plasma proteins, Predicted intracellular proteins, Transcription factors	Biological rhythms, Transcription, Transcription regulation	Activator, DNA-binding, Receptor	Cancer-related genes, Diabetes mellitus, Disease mutation, FDA approved drug targets, Obesity	Nucleoplasm, Vesicles
0.560219479	GAPDH	Glyceraldehyde-3-Phosphate Dehydrogenase	Enzymes, FDA approved drug targets, Plasma proteins, Predicted intracellular proteins	Apoptosis, Glycolysis, Translation regulation	Oxidoreductase, Transferase	FDA approved drug targets	Plasma membrane, Cytosol, Vesicles
0.465569273	NEU1	Neuraminidase 1	Disease related genes, Enzymes, Potential drug targets, Predicted intracellular proteins	Carbohydrate metabolism, Lipid degradation, Lipid metabolism	Glycosidase, Hydrolase	Disease mutation	Vesicles
0.456241427	NTRK1	Neurotrophic Receptor Tyrosine Kinase 1	Cancer-related genes, Disease related genes, Enzymes, FDA approved drug targets, Predicted membrane proteins, RAS pathway related proteins	Differentiation, Neurogenesis	Developmental protein, Kinase, Receptor, Transferase, Tyrosine-protein kinase	Cancer-related genes, Disease mutation, FDA approved drug targets, Proto-oncogene	Vesicles, Cytosol
0.438134431	MTOR	Mechanistic Target Of Rapamycin Kinase	Cancer-related genes, Disease related genes, Enzymes, FDA approved drug targets, Plasma proteins, Predicted intracellular proteins	Biological rhythms	Kinase, Serine/threonine-protein kinase, Transferase	Cancer-related genes, Disease mutation, Epilepsy, FDA approved drug targets, Mental retardation	Vesicles, Cytosol
0.427160494	LYN	LYN Proto-Oncogene, Src Family Tyrosine Kinase	Cancer-related genes, Disease related genes, Enzymes, Potential drug targets, Predicted intracellular proteins	Adaptive immunity, Host-virus interaction, Immunity, Inflammatory response, Innate immunity	Kinase, Transferase, Tyrosine-protein kinase	Cancer-related genes, Proto-oncogene	Golgi apparatus, Plasma membrane
0.421124829	CHUK	Component OfInhibitor Of Nuclear Factor Kappa B Kinase Complex	Disease related genes, Enzymes, Potential drug targets, Predicted intracellular proteins, RAS pathway related proteins		Kinase, Serine/threonine-protein kinase, Transferase		Nucleoplasm, Vesicles, Cytosol
0.417283951	TPI1	Triosephosphate Isomerase 1	Cancer-related genes, Disease related genes, Enzymes, Plasma proteins, Potential drug targets, Predicted intracellular proteins	Gluconeogenesis, Glycolysis	Isomerase, Lyase	Cancer-related genes, Disease mutation, Hereditary hemolytic anemia	Nucleoplasm, Vesicles
0.376680384	ATM	ATM Serine/Threonine Kinase	Cancer-related genes, Disease related genes, Enzymes, Plasma proteins, Potential drug targets, Predicted intracellular proteins, Predicted membrane proteins	Cell cycle, DNA damage	DNA-binding, Kinase, Serine/threonine-protein kinase, Transferase	Cancer-related genes, Disease mutation, Neurodegeneration, Tumor suppressor	Nucleoplasm, Vesicles
0.372565158	RUNX1	RUNX Family Transcription Factor 1	Cancer-related genes, Disease related genes, Plasma proteins, Predicted intracellular proteins, Transcription factors	Transcription, Transcription regulation	Activator, DNA-binding, Repressor	Cancer-related genes, Disease mutation, Proto-oncogene	Nucleoplasm, Vesicles
0.368998628	BSG	Basigin (Ok Blood Group)	Blood group antigen proteins, CD markers, Predicted intracellular proteins, Predicted membrane proteins, Transporters		Blood group antigen		Vesicles

**TABLE 5:** VarElect aggregated score obtained analyzing group 5 related to the central nervous system (G5a; 15 top-ranking genes).

Normalized score	Gene	Gene description	Protein class	Biological process	Molecular function	Disease involvement	Subcellular Location
1	TNF	Tumor Necrosis Factor	Cancer-related genes, Candidate cardiovascular disease genes, Disease related genes, FDA approved drug targets, Plasma proteins, Predicted intracellular proteins, Predicted secreted proteins		Cytokine	Cancer-related genes, FDA approved drug targets	
0.744887478	MAPT	Microtubule Associated Protein Tau	Candidate cardiovascular disease genes, Disease related genes, FDA approved drug targets, Plasma proteins, Predicted intracellular proteins			Alzheimer disease, Disease mutation, FDA approved drug targets, Neurodegeneration, Parkinsonism	Plasma membrane
0.64954711	APP	Amyloid Beta Precursor Protein	Disease related genes, FDA approved drug targets, Plasma proteins, Predicted intracellular proteins, Predicted membrane proteins, Transporters	Apoptosis, Cell adhesion, Endocytosis, Notch signaling pathway	Heparin-binding, Protease inhibitor, Serine protease inhibitor	Alzheimer disease, Amyloidosis, Disease mutation, FDA approved drug targets, Neurodegeneration	Golgi apparatus, vesicles
0.570314689	APOE	Apolipoprotein E	Cancer-related genes, Candidate cardiovascular disease genes, Disease related genes, Plasma proteins, Predicted secreted proteins	Cholesterol metabolism, Lipid metabolism, Lipid transport, Steroid metabolism, Sterol metabolism, Transport	Heparin-binding	Alzheimer disease, Amyloidosis, Cancer-related genes, Disease mutation, Hyperlipidemia, Neurodegeneration	Vesicles
0.534036791	TP53	Tumor Protein P53	Cancer-related genes, Disease related genes, Plasma proteins, Potential drug targets, Predicted intracellular proteins, Transcription factors, Transporters	Apoptosis, Biological rhythms, Cell cycle, Host-virus interaction, Necrosis, Transcription, Transcription regulation	Activator, DNA-binding, Repressor	Cancer-related genes, Disease mutation, Li-Fraumeni syndrome, Tumor suppressor	Nucleoplasm
0.530675133	IRF3	Interferon Regulatory Factor 3	Disease related genes, Predicted intracellular proteins, Transcription factors	Antiviral defense, Host-virus interaction, Immunity, Innate immunity, Transcription, Transcription regulation	Activator, DNA-binding	Disease mutation	Cytosol
0.530301615	PSEN1	Presenilin 1	Cancer-related genes, Disease related genes, Enzymes, Potential drug targets, Predicted intracellular proteins, Predicted membrane proteins, Transporters	Apoptosis, Cell adhesion, Notch signaling pathway	Hydrolase, Protease	Alzheimer disease, Amyloidosis, Cancer-related genes, Cardiomyopathy, Disease mutation, Neurodegeneration	Golgi apparatus, Nucleoplasm
0.519422915	SPTAN1	Spectrin Alpha, Non-Erythrocytic 1			Actin capping, Actin-binding, Calmodulin-binding	Disease mutation, Epilepsy, Mental retardation	Vesicles, Microtubules
0.503408348	SNCA	Alpha Synuclein	Disease related genes, Plasma proteins, Potential drug targets, Predicted intracellular proteins, Transporters			Alzheimer disease, Disease mutation, Neurodegeneration, Parkinson disease, Parkinsonism	
0.485059296	UNC93B1	Unc-93 Homolog B1, TLR Signaling Regulator		Adaptive immunity, Antiviral defense, Immunity, Innate immunity			Nucleoplasm
0.48440564	SOD1	Superoxide Dismutase 1	Cancer-related genes, Disease related genes, Enzymes, Plasma proteins, Potential drug targets, Predicted intracellular proteins		Antioxidant, Oxidoreductase	Amyotrophic lateral sclerosis, Cancer-related genes, Disease mutation, Neurodegeneration	Nucleoplasm, Plasma membrane
0.441777944	TBK1	TANK Binding Kinase 1	Disease related genes, Enzymes, Potential drug targets, Predicted intracellular proteins, RAS pathway related proteins	Antiviral defense, Host-virus interaction, Immunity, Innate immunity	Kinase, Serine/threonine-protein kinase, Transferase	Amyotrophic lateral sclerosis, Disease mutation, Glaucoma, Neurodegeneration	Nucleoplasm, vesicles
0.424596134	TGFB1	Transforming Growth Factor Beta 1	Cancer-related genes, Candidate cardiovascular disease genes, Disease related genes, Plasma proteins, Predicted intracellular proteins, Predicted secreted proteins		Growth factor, Mitogen	Cancer-related genes, Disease mutation	Golgi apparatus, Cytosol
0.420767579	ACADM	Acyl-CoA Dehydrogenase Medium Chain	Disease related genes, Enzymes, Potential drug targets, Predicted intracellular proteins	Fatty acid metabolism, Lipid metabolism	Oxidoreductase	Disease mutation	Mitochondria
0.41894668	TSC2	TSC Complex Subunit 2	Cancer-related genes, Disease related genes, Plasma proteins, Predicted intracellular proteins, Predicted membrane proteins	Host-virus interaction	GTPase activation	Cancer-related genes, Disease mutation, Epilepsy, Tumor suppressor	Cytosol

**TABLE 6:** VarElect aggregated score obtained analyzing group 5 related to the peripheral nervous system (G5b; 15 top-ranking genes).

Normalized score	Gene	Gene description	Protein class	Biological process	Molecular function	Disease involvement	Subcellular Location
1	NTRK1	Neurotrophic Receptor Tyrosine Kinase 1	Differentiation, Neurogenesis	Developmental protein, Kinase, Receptor, Transferase, Tyrosine-protein kinase	Cancer-related genes, Disease mutation, FDA approved drug targets, Proto-oncogene	Evidence at protein level	Vesicles, Cytosol
0.460741389	APOE	Apolipoprotein E	Cancer-related genes, Candidate cardiovascular disease genes, Disease related genes, Plasma proteins, Predicted secreted proteins	Cholesterol metabolism, Lipid metabolism, Lipid transport, Steroid metabolism, Sterol metabolism, Transport	Heparin-binding	Alzheimer disease, Amyloidosis, Cancer-related genes, Disease mutation, Hyperlipidemia, Neurodegeneration	Vesicles
0.457968476	MTOR	Mechanistic Target Of Rapamycin Kinase	Cancer-related genes, Disease related genes, Enzymes, FDA approved drug targets, Plasma proteins, Predicted intracellular proteins	Biological rhythms	Kinase, Serine/threonine-protein kinase, Transferase	Cancer-related genes, Disease mutation, Epilepsy, FDA approved drug targets, Mental retardation	Vesicles, Cytosol
0.378283713	COMT	Catechol-O-Methyltransferase	Disease related genes, Enzymes, FDA approved drug targets, Plasma proteins, Predicted intracellular proteins, Predicted membrane proteins	Catecholamine metabolism, Neurotransmitter degradation	Methyltransferase, Transferase	FDA approved drug targets, Schizophrenia	Vesicles
0.369819031	APP	Amyloid Beta Precursor Protein	Disease related genes, FDA approved drug targets, Plasma proteins, Predicted intracellular proteins, Predicted membrane proteins, Transporters	Apoptosis, Cell adhesion, Endocytosis, Notch signaling pathway	Heparin-binding, Protease inhibitor, Serine protease inhibitor	Alzheimer disease, Amyloidosis, Disease mutation, FDA approved drug targets, Neurodegeneration	Golgi apparatus, vesicles
0.319614711	SLC6A4	Solute Carrier Family 6 Member 4		Neurotransmitter transport, Symport, Transport		FDA approved drug targets	Golgi apparatus, Vesicles
0.299036778	PPARG	Peroxisome Proliferator Activated Receptor Gamma	Cancer-related genes, Disease related genes, FDA approved drug targets, Nuclear receptors, Plasma proteins, Predicted intracellular proteins, Transcription factors	Biological rhythms, Transcription, Transcription regulation	Activator, DNA-binding, Receptor	Cancer-related genes, Diabetes mellitus, Disease mutation, FDA approved drug targets, Obesity	Nucleoplasm, vesicles
0.288674839	LRRK2	Leucine Rich Repeat Kinase 2	Cancer-related genes, Disease related genes, Enzymes, Potential drug targets, Predicted intracellular proteins	Autophagy, Differentiation	GTPase activation, Hydrolase, Kinase, Serine/threonine-protein kinase, Transferase	Cancer-related genes, Disease mutation, Neurodegeneration, Parkinson disease, Parkinsonism	Nucleoplasm, Vesicles
0.26619965	TNFAIP3	TNF Alpha Induced Protein 3	Cancer-related genes, Disease related genes, Enzymes, Plasma proteins, Potential drug targets, Predicted intracellular proteins	Apoptosis, Inflammatory response, Ubl conjugation pathway	DNA-binding, Hydrolase, Multifunctional enzyme, Protease, Thiol protease, Transferase	Cancer-related genes, Disease mutation	Vesicles
0.260945709	SQSTM1	Sequestosome 1	Disease related genes, Predicted intracellular proteins	Apoptosis, Autophagy, Differentiation, Immunity		Amyotrophic lateral sclerosis, Disease mutation, Neurodegeneration	Vesicles, Cytosol
0.24270286	GAPDH	Glyceraldehyde-3-Phosphate Dehydrogenase	Enzymes, FDA approved drug targets, Plasma proteins, Predicted intracellular proteins	Apoptosis, Glycolysis, Translation regulation	Oxidoreductase, Transferase	FDA approved drug targets	Plasma membrane, Cytosol
0.214681845	DNM1L	Dynamin 1 Like		Biological rhythms, Endocytosis, Necrosis	Hydrolase	Disease mutation	Cytosol, Vesicles
0.193812026	TOR1A	Torsin Family 1 Member A	Disease related genes, Predicted intracellular proteins		Chaperone, Hydrolase	Disease mutation, Dystonia	Nuclear membrane, Vesicles
0.150175131	CAVIN1	Caveolae Associated Protein 1		Transcription, Transcription regulation, Transcription termination	RNA-binding, rRNA-binding	Congenital generalized lipodystrophy, Diabetes mellitus	Plasma membrane, Vesicles
0.140542907	LDHA	Lactate Dehydrogenase A			Oxidoreductase	Cancer-related genes, Disease mutation, FDA approved drug targets, Glycogen storage disease	Cytosol, Vesicles

Selected genes from aggregate ranks G124, G12345, and from single ranks G3, G5a, and G5b and subjected to the evaluation about the development of anti-COVID-19 pharmacology based on the repositioning of drugs already on the market (see section “Drug Repurposing Strategy”).

### Design of Drug Repositioning Strategy via Drug-Gene Interaction Data

The DrugBank repository^7^ (Wishart et al., 2018) was manually queried for the selection of drugs on the basis of their possible interference with the direct or indirect virus-host interaction. The criteria applied for selecting a restricted list of gene targets and the corresponding drugs were: (a) the highest place occupied in the aggregate VarElect ranks G124 (Table 2 and Supplementary Table S11) and G12345 (Table 3 and Supplementary Table S12) and in the single ranks G3, G5a, and G5b (Tables 4–6 and Supplementary Tables S13–S15); (b) the main cellular location of the target protein, selected on the basis of the possible virus-host interaction during cell entry (plasma membrane), RNA duplication (cytosol), RNA translation (endoplasmic reticulum), viral protein maturation and virus assembly (Golgi apparatus) and virus secretion (secretory vesicles); (c) the existence of approved drugs as suitable candidates for repositioning.

## Results

### Selection of Targets for Drug Repurposing

The application of the methodology detailed in section “Materials and Methods” leads to the selection of 260 target genes being potential candidates for drug repurposing. It turned out that out of these 260 genes, only 14 of them were ranked once (CDH1, CHEK2, TOP1, ADRB2, BIRC3, PRKAR1A, IKBKG, NEU1, CHUK, BSG, XPO1, WWOX, LDHA, and HSPA1A), while all of the others were repeated in two or more different ranks, with a total of 130 genes represented over 260 total entries in the pooled ranks. As for the main cellular locations, the majority of virus potential interactors were associated with cell nucleus (51), with less gene products located on plasma membrane and cytosol (15 each), Golgi/endoplasmic reticulum (12), vesicles (11), and mitochondria (7). The molecular function most represented was “enzyme” (46), while 16 activators/transcription factors, 9 membrane-bound receptors, 10 secreted proteins, 27 DNA-binding, 7 RNA-binding, 6 chaperones, 8 repressors were detected. Of note, 37 of the 130 unique gene targets were indicated in the Protein Atlas database as generic Virus-Host interactors, while 8 genes codify for proteins with antiviral activities. Finally, the analysis of “protein class” fields in Supplementary Tables S11–S15, revealed that 65 out of 130 genes were previously identified as non-COVID-19 specific potential drug targets, yet subjected to evaluation or approved by Regulatory Agencies (FDA and EMA).

### Drug Repurposing Strategy

Following our extensive, multi-level analysis, we identified high ranking genes that may be potential pharmacological targets, fulfilling the requirements for a fast and safe drug repositioning strategy (Table 7). Most of them are enzymes (kinases, phosphatases, etc., i.e., AKT1, CDK4, LYN, MAPK14, TBK1, CHEK2, ATM, LRRK2, CHUK, SRC, MTOR, and MAPK1) belonging to various downstream signaling pathways, or involved in essential cell physiological processes, such as DNA replication, RNA synthesis and translation (i.e., RANBP2, SMARCA4, FUS, XPO1, DDX58, CAVIN1, and IFIH1), protein processing (i.e., PLAT, CASP8, PSEN1, APP, CASP3, XIAP, SERPING1, and TNFAIP3) energy consumption (i.e., GLA, NEU1) metabolic pathways (i.e., LDHA, WWOX, HMOX1, SDHB, SDHA, HADHA, ACADM, SOD1, GAPDH, PLOD1, and NOS2). Few proteins belongs to the class of secreted factors (i.e., PLAT, FBN1, TGFB1, SPP1, TNF, SERPING1, APOE, C4A, FN1, and TNFRSF1A). Some of the identified targets, based on their cellular compartmentalization, most probably may be activated/repressed in the process of virus entry and replication or viral proteins post-translational processing (i.e., HMOX1, APP, LYN, XIAP, SOD1, HIF1A, EGFR, ICAM1, FAS, CD209, CDH1, SRC, FLNA, DDX58, MAPT, CTNNB1, ERBB2, ADRB2, GAPDH, HSPB1, and CAVIN1), or may interact with virus proteins during the last phase of virus secretion through secretory vesicles (i.e., DNM1L, LDHA, NKX2-1, SLC6A4, CAVIN1, APOE, TNFAIP3, COMT, NEU1, BSG, SCARB1, MTOR, SQSTM1, NTRK1, and SPTAN1). A list of 18 potentially effective pharmacological targets with associated approved drugs, is presented in Table 7. Such genes have been selected prioritizing the existence of an already approved, safe and effective pharmacology. Then, gene candidates that were not considered as directly involved in virus-host protein interactions were discarded, i.e., those located in cell nucleus structures or those involved in essential, redundant and/or non-targetable cell metabolic/physiologic processes. Finally, all potential (and strong) candidates already under clinical investigation as potential drug targets for COVID-19 pandemics (i.e., TNF, highest in more than one aggregate rank in the VarElect analysis) were also excluded. The resulting list encompass plasma membrane receptors (i.e., EGFR, ERRB2, FGFR1, among others), proteins mainly localized in the Golgi and endoplasmic reticulum (CALR, APP, LYN, and COMT), Cytosol (LDHA, MTOR), vesicles (TBK1, COMT, APP, LDHA, and MTOR). Some of the proposed genes are potentially targeted by the same or similar drugs (as evidenced in the “potential alternative targets” fields in Table 6 drugs). Moreover, some of the proposed drugs are potentially effective on pharmacological targets already identified as potential drug targets or under investigation in ongoing clinical trials on COVID-19 patients (i.e., VEGFA, C1QA, C1QB, and C1QC)^8^. All of the selected genes were relatively high in their aggregated ranks (see Tables 2–6).

**TABLE 7:** Summary of drugs potentially relevant for COVID-19 chosen via the data-driven drug repositioning strategy.

Drug(s)	DrugBank ID	Target gene	Possible compartment for Target-SARS-Cov-2 interaction	COVID-19-related phase	Drug description	Status	Approved conditions	Potential alternative targets	Reference/Note
Afatinib	DB08916	EGFR	Plasma membrane	Virus entry	Small Molecule Inhibitor	Approved	Metastatic Non-Small Cell Lung Cancer, Refractory, metastatic squamous cell Non-small cell lung cancer	ERBB2, ERBB4	
		ERBB2	Plasma membrane	Virus entry	Small Molecule, Inhibitor	Approved	Metastatic non-small cell lung cancer (NSCLC) with common epidermal growth factor receptor (EGFR) mutations	EGFR, ERBB4,	https://www.boehringer-ingelheim.us/press-release/fda-approves-new-indication-gilotrif-egfr-mutation-positive-nsclc
Artenimol	DB11638	FLNA	Plasma membrane	Virus entry	Small Molecule	Approved, investigational	Antimalarial agent	ANXA2, CAST, DPYSL2, DSP, HSPB1, IQGAP1, MAP4, RPL4, RPL10, RPL13, RPL14, RPL35, RPL17, RPL18, RPL19, RPL23A,	https://apps.who.int/iris/bitstream/handle/10665/162441/9789241549127_eng.pdf;jsessionid=F0093888/B29AE0/CE2AF698/13FFCFF6FD?sequence=1
Bosutinib	DB06616	LYN	Golgi apparatus, Plasma membrane	Virus entry, virus assembly	Small Molecule	Approved	Philadelphia chromosome-positive (Ph+) chronic myelogenous leukemia (CML)	BRC, ALB1, HCK, SRC, CDK2, MAP2K1, MAP2K2, MAP3K2, CAMK2G	https://pubmed.ncbi.nlm.nih.gov/23674887/
		SRC	Plasma membrane	Virus entry	Small Molecule	Approved	Inhibitor for the treatment of Philadelphia chromosome-positive (Ph+) chronic myelogenous leukemia (CML)	LYN, HCK, CDK2, MAP2K1, MAP2K2, MAP3K2, CAMK2G, ABL1, BCR,	https://pubmed.ncbi.nlm.nih.gov/23098112/
Calcium Phosphate	DB11348	CALR	Endoplasmic reticulum	Viral protein synthesis	Small Molecule	Approved	Calcium and phosphate supplement, antacid,	CASR, CIB1, SRI, CHP1, CANX, FBN2, S100B, CASQ2, RGN, PEF1, S100A6, TPT1, CIB2, CALM1, FBN3	
Calcium phosphate dihydrate	DB14481	CALR	Endoplasmic reticulum	Viral protein synthesis	Small Molecule	Approved	Counter calcium and phosphate supplement, antacid	CASR, PDCD6, SPARC, CANX, S100A6, TPT1, CIB2, FBN2, SRI, S100B, CASQ2, RGN, PEF1, CHP1,	
Carboplatin	DB00958	SOD1	Nucleoplasm, Plasma membrane	Virus entry	Small Molecule	Approved	Antineoplastic activity	XDH, MPO, GSTT1, GSTM1, GSTP1, NQO1, MT1A, MT2A	https://patents.google.com/patent/US7259270?oq=07259270
Cetuximab	DB00002	EGFR	Plasma membrane	Virus entry	Monoclonal Antibody, Antagonist	Approved	Metastatic Colorectal Cancer	FCGR3B, C1QA, C1QB, C1QC, FCGR3A, FCGR1A, FCGR1B	http://www.ncbi.nlm.nih.gov/pubmed/11408594
Cisplatin	DB00515	SOD1	Nucleoplasm, Plasma membrane	Virus entry	Small Molecule	Approved	Sarcomas, small cell lung cancer, ovarian cancer, lymphomas and germ cell tumors	MPG, A2M, TF, ATOX1	
Docetaxel	DB01248	MAPT	Plasma membrane	Virus entry	Small Molecule	approved, investigational	Anti-mitotic chemotherapy for breast, ovarian, non-small cell lung, androgen independent metastatic prostate, gastric adenocarcinoma and head and neck cancer.	TUBB1, BCL2, MAP2, MAP4, NR1I2, CYP3A4,	https://pubmed.ncbi.nlm.nih.gov/18068131/
Entacapone	DB00494	COMT	Endoplasmic reticulum, vesicles	Viral protein synthesis, virus release	Small Molecule, Inhibitor	Approved	Parkinson’s disease		http://www.ncbi.nlm.nih.gov/pubmed/11440283
Everolimus	DB01590	MTOR	Vesicles, Cytosol	Virus replication and release	Small Molecule	Approved	Immunosuppressant to prevent rejection of organ transplants		
Ferric derisomaltose	DB15617	TFRC	Plasma membrane	Virus entry	Small Molecule	Approved	Anemia, non-hemodialysis dependent chronic kidney disease	HBA1	https://www.accessdata.fda.gov/drugsatfda_docs/label/2020/208171s000lbl.pdf
Fostamatinib	DB12010	TBK1	Nucleoplasm, vesicles	Virus release	Small Molecule	Approved, investigational	Rheumatoid Arthritis and Immune Thrombocytopenic Purpura (ITP)	CTSL, ABL1, RPS6KA6, MET, TEK, TGFBR1, TGFBR2, SYK,	https://www.fda.gov/drugs/resources-information-approved-drugs/fda-approves-fostamatinib-tablets-itp
Lansoprazole	DB00448	MAPT	Plasma membrane	Virus entry	Small Molecule	approved, investigational	Ulcerative, gastroesophageal reflux disease (GERD), and other pathologies caused by excessive acid secretion	ATP4A	https://pubmed.ncbi.nlm.nih.gov/19006606/
Natalizumab	DB00108	ICAM1	Plasma membrane	Virus entry	Monoclonal Antibody	Approved, Investigational	Multiple sclerosis	ITGA4, FCGR3B, FCGR1A	Natalizumab was voluntarily withdrawn from United States market because of risk of Progressive multifocal leukoence-phalopathy (PML). It was returned to market July, 2006
		CD209	Plasma membrane	Virus entry	Monoclonal Antibody	Approved, Investigational	Multiple sclerosis	FCGR1A, ITGA4, ICAM1, FCGR3B	Natalizumab was voluntarily withdrawn from United States market because of risk of Progressive multifocal leukoencephalopathy (PML). It was returned to market July, 2006
Nintedanib	DB09079	LYN	Golgi apparatus, Plasma membrane	Virus entry, virus assembly	Small Molecule	Approved	Pulmonary fibrosis, systemic sclerosis-associated interstitial lung disease, and non-small cell lung cancer	KDR, LCK, SRC, PDGFRA, PDGFRB, FGFR1, FGFR2, FGFR3, FLT1, FLT3, FLT4,	https://www.accessdata.fda.gov/drugsatfda_docs/label/2018/205832s010lbl.pdf
		SRC	Plasma membrane	Virus entry	Small Molecule	Approved	Pulmonary fibrosis, systemic sclerosis-associated interstitial lung disease, and non-small cell lung cancer (NSCLC)	FLT1, KDR, FLT4, PDGFRA, PDGFRB, FGFR1, FGFR2, FGFR3, FLT3, LCK, LYN,	https://www.accessdata.fda.gov/drugsatfda_docs/label/2018/205832s010lbl.pdf
		FGFR1	Plasma membrane	Virus entry	Small Molecule	Approved	Pulmonary fibrosis, systemic sclerosis-associated interstitial lung disease, and non-small cell lung cancer (NSCLC)	FLT1, KDR, FLT4, PDGFRA, PDGFRB, FGFR1, FGFR2, FGFR3, FLT3, LCK, LYN,	https://www.accessdata.fda.gov/drugsatfda_docs/label/2018/205832s010lbl.pdf
Osimertinib	DB09330	EGFR	Plasma membrane	Virus entry	Small Molecule Inhibitor	Approved	Metastatic Non-Small Cell Lung Cancer		http://www.ncbi.nlm.nih.gov/pubmed/26522274
Palifermin	DB00039	FGFR1	Plasma membrane	Virus entry	Small Molecule	Approved	Oral mucositis	FGFR2, NRP1, FGFR4, FGFR3, HSPG2,	
Pertuzumab	DB06366	ERBB2	Plasma membrane	Virus entry	Monoclonal antibody, Inhibitor	Approved	Metastatic HER2-positive breast cancer.		https://www.accessdata.fda.gov/drugsatfda_docs/label/2020/125409s124lbl.pdf
Regorafenib	DB08896	FGFR1	Plasma membrane	Virus entry	Small Molecule	Approved	Metastatic colorectal cancer and advanced gastrointestinal stromal tumors	FLT1, KDR, FLT4, KIT, PDGFRA, PDGFRB, FGFR2, DDR2, EPHA2, RAF1, BRAF, MAPK11, FRK, ABL1, RET, TEK, NTRK1	
Stiripentol	DB09118	LDHA	Cytosol, Vesicles	virus replication	Small Molecule	Approved	Anticonvulsant drug used in the treatment of epilepsy	LDHB, GABA(A) Receptor (Protein Group)	https://www.accessdata.fda.gov/scripts/cder/daf/index.cfm?event=reportsSearch.process
Temsirolimus	DB06287	MTOR	Vesicles, Cytosol	Virus replication and release	Small Molecule	Approved	Renal cell carcinoma (RCC)		
Tromethamine	DB03754	APP	Plasma membrane, Golgi apparatus, vesicles	Virus entry, virus assembly	Small molecule, Inhibitor	Approved	Prevention and correction of metabolic acidosis		http://www.ncbi.nlm.nih.gov/pubmed/8380642
Urea	DB03904	CTNNB1	Plasma membrane	Virus entry	Small Molecule	Approved, Investigational		ARG1, CA2, yedY, DHFR	
Vandetanib	DB05294	EGFR	Plasma membrane	Virus entry	Small Molecule Inhibitor	Approved	Non-resectable, locally advanced, or metastatic medullary thyroid cancer	VEGFA	

## Discussion

In this work, we identified and prioritized a number of target genes involved in different ways in the host SARS-CoV-2 invasion and response via a network proximity-based procedure. Subsets of such target genes were subsequently identified in different organs and systems of the human organism, with the aim of isolating and classifying, in functionally coherent tissue/organ groups (respiratory and digestive epithelia, blood, filter/excretory tissues, and nervous system), the mostly suited target genes for the development of a pharmacology based on the repositioning of drugs already on the market. For each group of tissues, relevant target classifications have been established, on the basis of the potentially associated pathological phenotypes, previously described as characterizing the COVID-19 disease (Adhikari et al., 2020). The highest target genes in the individual tissue ranking were then grouped to reach the selection of 130 unique targets, 90% of which were significant in two or more of the tissues considered. Finally, by analyzing each relevant target, a pharmacological proposal has been defined for 18 target genes and expected to interfere with the virus-host interaction in the various infectious phases and the viral replication cycle.

Computationally based approach has been already considered for drug repurposing: for example Zhou et al. (2020) prioritize sixteen potential repurposable drugs against SARS-CoV-2 using a network proximity analysis. In particular, the authors mapped the drug-target network into a selected COVID-19 host interactome to search for cellular target; (Cheng et al., 2020) proposed a combination of anti-inflammatory and antiviral therapeutics using a network based approach in which proximity measure quantifies the relationship between COVID-19 disease modules and drug targets in the Human PPIs network. Our computationally driven approach revealed that it is possible to hypothesize unequivocal and functional pharmacological interventions to counteract the development of symptoms affecting various organs and systems. This consideration arises from the evidence that some of the pharmacological targets identified (i.e., EGFR, ERBB2, APP, ICAM1, and FAS), may be important to prevent the interaction of the virus with the cell surface in different target organs. However, it is also necessary to conceive pharmacological strategies based on the combination of different drugs, able to counter, by targeting different players of the virus-host interaction, the various stages through which the infection develops at the cellular level (virus entry, replication, viral protein processing, and release of new virus). Finally, the association of therapies interfering with virus-host interaction with strategies aimed at bringing back under control the inflammatory phenomena, with which the body fights the infection and which have often proved fatal (Astuti and Ysrafil, 2020), is deserved.

Computational criteria and methods brought to the definition of COVID-19 proximal target genes. Then, biological criteria lead to select the relevant interactions, potential targets for drug repurposing, associated with different stages of viral infection and the development of the constellation of symptoms already described in COVID-19 patients (Adhikari et al., 2020; Ahmad and Rathore, 2020). Virus-host interactions may stand as physical interactions between viral and human proteins or as indirect interactions based on the triggering, after virus challenge, of the complex network of metabolic processes characterizing eukaryotic cells. In the analysis presented in this work, in addition to the classifications of relevant target genes, their cellular localization was also taken into consideration, with the aim of hypothesizing possible specific interactions for the individual compartments of the cell, in which the viral proteins could relate with human ones. Based on such rationale, plasma membrane-bound proteins have been considered as alternative interactors for virus entry. Cytoplasm-located proteins may conceivably interact with the virus during its replication phase, while endoplasmic reticulum and Golgi proteins could interact with the viral M protein and the viral proteins post-translational processing (Astuti and Ysrafil, 2020). Finally, vesicles-associated interactors have been hypothesized to play a role in the virus secretion.

It is known that the receptor-binding domains on the SARS-CoV-2 S protein bind with high affinity to human ACE2 (Wrapp et al., 2020), an interaction accounting for virus entry in the host cell and for its transmissibility. The analysis of COVID-19 extended interactome indicates several membrane bound gene/proteins (i.e., ICAM1, EGFR, ERBB2, APP, ADR2, FAS, CDH1, and MAPT), whose activity and/or expression could be affected by SARS-CoV-2 challenge. Evidence for alternative interaction of virus S protein with receptors other than ACE2 have been not only already suggested by computational analysis (Milanetti et al., 2020), but also demonstrated in vitro (Ulrich and Pillat, 2020). Furthermore, some of the selected proteins could also account for additional host interactions, not necessarily related with the transmission of disease. RNA-binding proteins present in the cytosol, part of the extended interactome and with a high position in the VarElect ranks (i.e., RANBP2, XPO1, and CDKN2A), could reasonably participate in the replication and translation phases of the viral RNA. Similarly, proteins associated with the endoplasmic reticulum and Golgi membranes (i.e., CALR, COMT, CAV1, and PTCH1) could be involved in the translation processes of the viral RNA and in the subsequent protein processing. Lastly, it is worth mentioning the interactions foreseen by computational analysis with secreted proteins. Among the most important are those with TNF, which plays a central role in the cytokine storm that characterizes the most severe phase of the disease, and which already constitutes a drug target challenged in intensive care units worldwide.

There are actually dozens of drug targets tested for COVID-19 in more than 1200 clinical trials worldwide, as reported in the DrugBank repository (see text footnote 8). Among these, only TNF has been identified by our analysis as being part of the COVID-19 host target genes. Recently, a list of more than 300 possible target genes has been experimentally observed to interact with Sars-CoV-2 proteins and thus considered for the development of anti-COVID, repositioning-based therapies (Gordon et al., 2020), of which only 11 (NEU1, SCARB1, TBK1, COMT, HMOX1, FBN1, GLA, ACADM, DNMT1, PLAT, and TOR1A) are shared with those predicted through the methodology applied in the present work after a data-driven prioritization. In addition, despite the apparent abundance of potential pharmacological targets proposed through data analysis, relatively few of these lend themselves to being used in drug repositioning strategies. The final data of the present work, summarized in the Table 6, indicate that among the potential first 130 targets identified, because at the top positions in the ranks of potential efficacy elaborated through our methodology, only 18 preliminary appear as suitable candidates for drug repositioning. The reasons lie in the lack, for most of the ranked genes, of pharmacologically active drugs already approved by the Regulatory Agencies, or in the impossibility of developing, for many of them participating in essential processes in cellular physiology, a pharmacological approach that modifies their activity, or, finally, in the difficulty of using drugs with a significant impact on physiology or with a high risk of inducing side effects in patients already deeply debilitated by SARS-CoV-2 infection.

## Conclusion and Perspectives

The pandemic caused by SARS-CoV-2 represents an open and unresolved challenge for the global health system. The need to identify drugs that demonstrate efficacy in countering both the mechanisms of interaction of SARS-CoV-2 with host cells and to control the devastating inflammatory phenomena that characterize the late stages of viral infection, requires increasingly urgent answers. The biomedical research approach based on the repurposing of already approved drugs seems to be one of the most viable strategies in this struggle. This work, via a data-driven network-based procedure, provides a viable and alternative drug repurposing strategy to be considered for clinical trial. The proposed approach has been conceived to support the comprehension of the molecular landscape of COVID-19 as well as the identification of genes that are not immediately associated to SARS-CoV-2 invasion, or not taken into consideration in respect to the host defense regulation and dynamics, and may thus suggest new directions for further studies and analyses. We leave open the possibility of extending our preliminary analysis by increasing the number of genes present in the currently proposed COVID-19 proximal target genes and/or by extending the selection of potential target genes identified through functional analysis to a greater number than the current one. Under the computational point of view further approaches could be considered, for instance several network topological measures and/or a combination of them could be considered to select COVID-19 proximal candidate target genes and to investigate whether/how changes in the drugs proposal occur.

## Data Availability Statement

All datasets presented in this study are included in the article/Supplementary Material.

## Author Contributions

PT conceived the study. All authors collected the data, ran the analysis, wrote the manuscript, and approved the submitted version.

## Conflict of Interest

The authors declare that the research was conducted in the absence of any commercial or financial relationships that could be construed as a potential conflict of interest.

## Footnotes

1 Source: COVID-19 Dashboard by the Center for Systems Science and Engineering (CSSE) at Johns Hopkins University (JHU), available at https://gisanddata.maps.arcgis.com/apps/opsdashboard/index.html#/bda7594740fd40299423467b48e9ecf6, retrieved August 26th, 2020.

2 Source: https://www.drugbank.ca/Covid-19, retrieved July 6th, 2020.

3 https://wiki.thebiogrid.org/doku.php/covid

4 www.proteinatlas.org

5 Wadman M. et al., “How does coronavirus kill? Clinicians trace a ferocious rampage through the body, from brain to toes”. doi: 10.1126/science.abc3208

6 https://www.proteinatlas.org/about/assays+annotation#rna

7 https://www.drugbank.ca/

8 https://www.drugbank.ca/covid-19#drug-targets

## Supplementary Material

Supplementary File 1 Cytoscape session file (.cys) with human interactome data.

Supplementary Table 1 List of the 500 COVID-19 associated host genes derived from the BioGRID dataset (BIOGRID-CORONAVIRUS-3.5.186.tab3.zip).

Supplementary Table 2 Output of the DIAMOnD algorithm: first 1000 genes ranked for connectivity significance starting from the 500 COVID-19 associated seed genes.

Supplementary Table 3 Output of the network propagation/heat diffusion algorithm run with 5 different diffusion times starting from the 500 COVID-19 associated seed genes.

Supplementary Table 4 Aggregated chart of the first 1500 genes ranked for COVID-19 proximity.

Supplementary Tables 5–10 VarElect scores obtained for genes in the COVID-19 extended interactome expressed in different groups of tissues/organs (reported in each Table) and selected for their association with disease phenotypes (reported in each Table) specifics for each tissue/organ.

Supplementary Tables 11–15 Aggregated and normalized VarElect scores for the first 100 (G124 and G12345) and 20 (G3, G5a, and G5b) genes in the VarElect analysis depicted in Supplementary Tables 5–10.
